# Melatonin or its analogs as premedication to prevent emergence agitation in children: a systematic review and meta-analysis

**DOI:** 10.1186/s12871-023-02356-x

**Published:** 2023-11-30

**Authors:** Dongni Zhang, Xiaotong Jia, Duomao Lin, Jun Ma

**Affiliations:** Department of Anesthesiology, Beijing Anzhen Hospital, Capital Medical University, Beijing, 100029 China

**Keywords:** Emergence agitation, Melatonin, Pediatrics

## Abstract

**Background:**

Emergence agitation (EA) is a prevalent complication in children following general anesthesia. Several studies have assessed the relationship between melatonin or its analogs and the incidence of pediatric EA, yielding conflicting results. This meta-analysis aims to assess the effects of premedication with melatonin or its analogs on preventing EA in children after general anesthesia.

**Methods:**

PubMed, EMBASE, the Cochrane Library, ProQuest Dissertations & Theses Global, Web of Science, CNKI, Wanfang Data, clinicaltrials.gov, and WHO International Clinical Trials Registry Platform were searched until 25 November 2022. We included randomized controlled trials that assessed EA in patients less than 18 years old who underwent general anesthesia. We excluded studies that did not use a specific evaluation to assess EA.

**Results:**

Nine studies (951 participants) were included in this systematic review. Melatonin significantly reduced the incidence of EA compared with placebos (risk ratio 0.40, 95% CI 0.26 to 0.61, *P* < 0.01) and midazolam (risk ratio 0.48, 95% CI 0.32 to 0.73, *P* < 0.01). Dexmedetomidine remarkably decreased the incidence of EA compared with melatonin (risk ratio 2.04, 95% CI 1.11 to 3.73, *P* = 0.02).

**Conclusions:**

Melatonin premedication significantly decreases the incidence of EA compared with placebos and midazolam. Dexmedetomidine premedication has a stronger effect than melatonin in preventing EA. Nevertheless, further studies are warranted to reinforce and validate the conclusion on the efficacy of melatonin premedication in mitigating EA in pediatric patients.

**Supplementary Information:**

The online version contains supplementary material available at 10.1186/s12871-023-02356-x.

## Introduction

Emergence agitation (EA) is a prevalent complication in children after general anesthesia with a reported incidence of 10–80% [1]. Characterized by perceptual disturbances and psychomotor agitation, EA may be distressing for children and their parents as it delays wound healing and prolongs the length of the hospital stay [2]. A lack of premedication is a risk factor for the development of EA [2], and pharmacological premedication is considered effective in preventing EA [3].

Melatonin, a neurohormone secreted by the pineal gland in the human brain, has several important physiological functions [4]. Low serum melatonin levels have been demonstrated to be associated with delirium in adult patients [5]. Melatonin has been implicated as having anti-inflammatory [6], anxiolytic [7], and analgesic properties [4], which may reduce the precipitating factors of EA [1]. Exogenous melatonin is considered beneficial in various pediatric therapies due to its high therapeutic safety and few adverse effects [8]. Recently, researchers have paid increasing attention to melatonin premedication for children undergoing general anesthesia. Several studies have evaluated melatonin and the incidence of EA in children, with conflicting results. Some studies reported that melatonin, compared with placebos or midazolam, significantly reduced EA [7, 9], and one study demonstrated that dexmedetomidine had a stronger effect than melatonin in reducing EA [10]. Meanwhile, another study comparing melatonin with clonidine and dexmedetomidine found no significant differences [11].

In the context of melatonin’s limited oral bioavailability and short half-life, scientists have developed several melatonin analogs to improve its duration of action, bioavailability, and receptor affinity [12]. Notable among these analogs are ramelteon, tasimelteon, and agomelatine. Specifically, ramelteon, a tricyclic synthetic analog, selectively targets melatonin receptor 1 and melatonin receptor 2 [13]. Studies have confirmed that ramelteon has a superior receptor affinity and longer half-life than melatonin [13]. Ramelteon has demonstrated efficacy in reducing delirium risk among hospitalized adult patients [14]. Furthermore, in pediatric surgical settings, a clinical trial has explored ramelteon as a premedication to assess its potential to mitigate EA [15].

A meta-analysis [16] in 2015 reported that melatonin premedication could prevent EA in children who had undergone general anesthesia. However, as it only included four studies the authors could not draw conclusions on the comparison between melatonin and midazolam or dexmedetomidine and did not assess publication bias. In addition, this meta-analysis [16] did not include studies on melatonin analogs, which have been used to prevent EA in children [15]. In light of the inconsistent results of recent studies [9–11], there is a need to update the association between premedication with melatonin or its analogs and the incidence of pediatric EA.

Thus, we performed a comprehensive updated meta-analysis and systematic review to evaluate the effects of premedicated melatonin or its analogs in preventing EA in children who have undergone general anesthesia.

## Method

This meta-analysis was conducted according to the PRISMA guidelines [17]. The study protocol was prospectively registered with PROSPERO (registration no. CRD42022355915).

### Eligibility criteria

The following inclusion criteria were adopted: (1) Patients less than 18 years old who underwent general anesthesia; (2) Prophylactic use of melatonin or its analogs, including ramelteon, tasimelteon, and agomelatine; (3) Use of placebos or alternative premedication drugs as control; (4) Outcomes including the assessment of EA or emergence delirium; and (5) Randomized controlled trials (RCTs).

The following studies were excluded from the systematic review: (1) Those not assessing EA using a specific evaluation tool; (2) Case reports, reviews, editorial letters, and animal studies; (3) Ongoing clinical trials; and (4) Redundant publications and repeated studies from the same trial.

### Search strategy

Relevant research published until 25 November 2022 was searched on MEDLINE (PubMed), Excerpta Medica Database (EMBASE), Web of Science, the Cochrane Library, ProQuest Dissertations & Theses Global, China National Knowledge Infrastructure (CNKI), and Wanfang Data. Study registrations published in the WHO International Clinical Trials Registry Platform and ClinicalTrials.gov were also searched. We searched Google Scholar to identify gray literature and checked the first 300 results [18]. The references of included studies were also hand-searched. No language restriction was applied. The search strategy for the electronic databases is demonstrated in eTable 1 in the Supplement. Two authors (D.Z. and X.J.) independently screened the title and abstract of each study to identify eligible studies. Potential eligible studies were retrieved as full-text studies. Any discrepancy was resolved through discussion with a third author (D.L.).

### Outcomes

The primary outcome was the incidence of EA, comparing melatonin and its analogs against controls, including placebos and other premedication types. The secondary outcome was the incidence of the adverse effects of premedication.

### Data extraction

Two investigators (D.Z. and X.J.) independently reviewed the retrieved studies and extracted relevant data using the data extraction tables. Discrepancies between the extractions were resolved through discussion. Data such as author, publication date, sample size, age, gender, type of surgery, American Society of Anesthesiologists (ASA) physical status, anesthetic agents, EA diagnosis tool, administration mode, given dose of melatonin, type of control, postoperative analgesia, EA incidence rate, adverse effects of premedication, and funding sources were collected. If any of these data were missing, the corresponding authors were contacted through email. For studies with multiple dosages of an alternative premedication drug, the group with the recommended dose was selected as the control group (e.g., midazolam at least 0.5 mg kg^− 1^ [19, 20] in children below 10 years old). When the multiple-time-points EA incidence was reported without the total EA incidence across various groups, the closest time point after emergence was selected to collect data. For studies with multiple melatonin dosage groups, all melatonin groups were combined into a single group.

### Evaluating the studies’ risk of bias

Two investigators (D.Z. and X.J.) independently evaluated the risk of bias using the Cochrane Collaboration Risk of Bias Tool (Version 2.0) [21]. Each study was categorized as low risk, some concerns, or high risk. Any disagreement between the reviewers was resolved through consensus or discussion with a third reviewer (D.L.).

### Statistical analysis

Any studies with a high risk of bias were excluded from the meta-analysis. Data were expressed using the pooled risk ratio (RR) with a 95% confidence interval (CI). Heterogeneity between studies was evaluated using an *I*^*2*^ test. The *I*^*2*^ value of 0% corresponded to no degree and 25% to low, 50% to moderate, and 75% to high degrees of heterogeneity. The pooled effect size was measured by a random effects model (DerSimonian and Laird) if *I*^*2*^ was larger than 50%; otherwise, a fixed effects model (Mantel–Haenszel) was employed. A continuity correction of 0.5 was applied if zero events were reported in one group. When heterogeneity was observed, a subgroup analysis was performed to further identify heterogeneity sources by type of melatonin (melatonin vs. ramelteon). Trial sequential analysis (TSA) was performed to calculate the adjusted CI and quantify the required information size (RIS) and monitoring boundaries. In the TSA model, the type I and type II error rates were 5% and 20%, respectively. Relative risk reduction was defined as 25% [16], and the incidence of the control arm was calculated based on the overall incidence of the corresponding control groups. Meta-regression was used to test the dose–response associations. Visual inspection of asymmetry in the funnel plots was applied to evaluate the publication bias. Statistical tests were conducted with Stata Version 17. TSA was carried out using TSA Viewer Version 0.9 Beta (www.ctu.dk/tsa). *P* < 0.05 was deemed significant.

### Grading of the evidence

The Grading of Recommendations Assessment, Development, and Evaluation (GRADE) guidelines (GRADEpro software; http://gradepro.org/) were used to judge the certainty of the evidence. Certainty was initially assessed as high and downgraded or upgraded according to the risk of bias, imprecision, indirectness, inconsistency, publication bias, and dose-response gradient. The certainty of the evidence was defined as very low, low, moderate, or high.

## Results

### Study selection

**Fig. 1:**
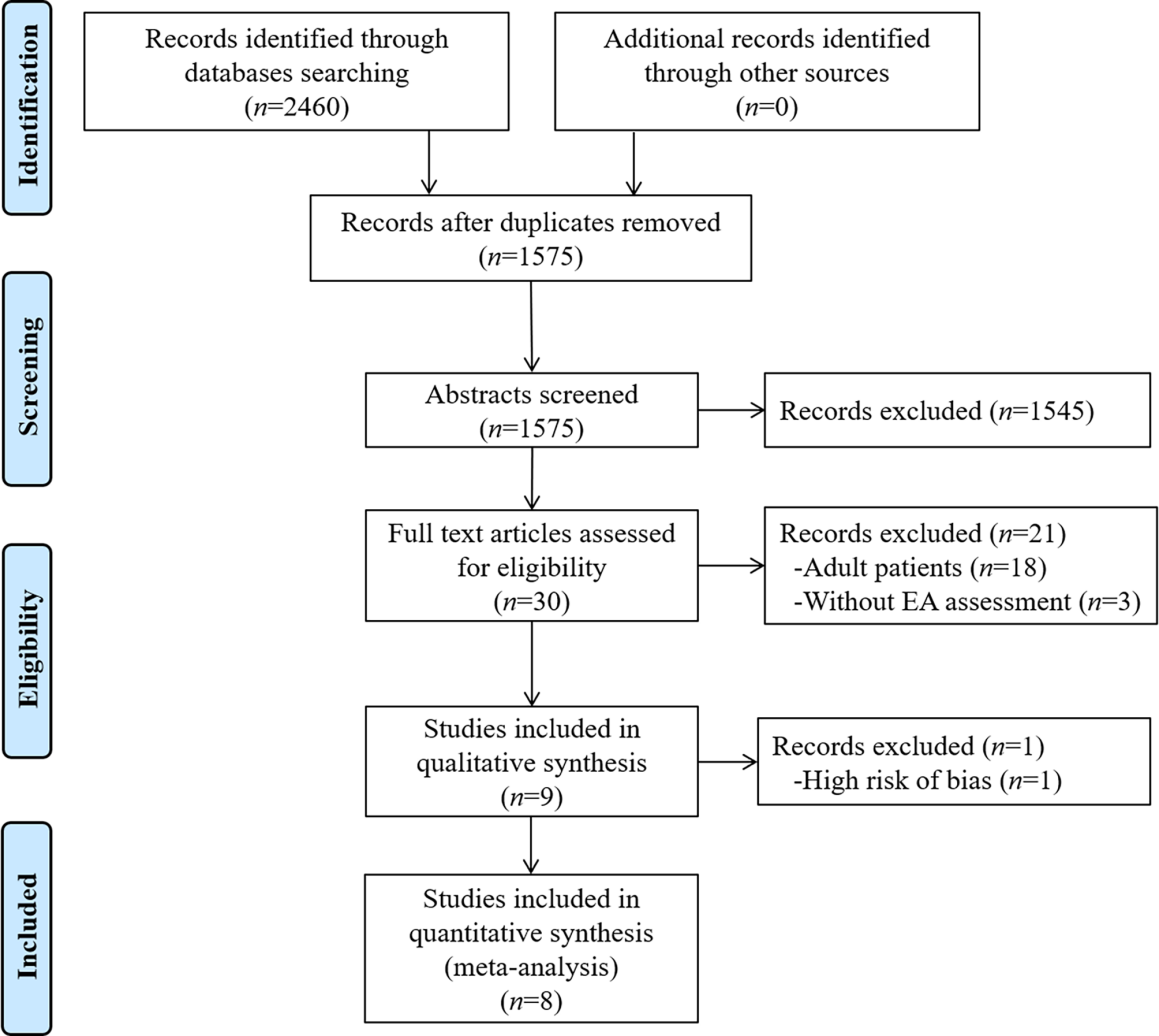
Flow diagram of the literature search and study selection

A flow diagram of the search and selection processes is shown in Fig. 1. In total, 2460 studies were identified from the databases and other sources. After duplicates were excluded (*n* = 885), 1575 studies were subjected to title and abstract screening, and 30 full-text studies were then evaluated for eligibility. Twenty-one further studies were excluded due to considering adult patients (*n* = 18) or lacking an EA assessment (*n* = 3). Nine studies were subjected to qualitative analysis. After one study with a high risk of bias was excluded, eight studies were included in this meta-analysis.

### Study characteristics

The study characteristics are shown in Table 1. A total of 951 participants was included in nine RCTs evaluating the preventive effects of melatonin premedication on pediatric EA [7, 9–11, 15, 22–25]. Eight studies were peer-reviewed publications [7, 9–11, 15, 22–24], while one study was an unpublished master’s thesis [25]. The sample size ranged from 48 to 163, and the children ranged in age from 1.5 to 9 years old. Eight studies used oral administration of 0.05 to 0.5 mg kg^− 1^ melatonin, and one study used a melatonin analog (ramelteon 0.1 mg kg^− 1^) [15]. The comparators included placebos [7, 9, 15, 23–25], midazolam [7, 9, 22, 24, 25], dexmedetomidine [10, 11, 24], and clonidine [11]. Anesthesia was induced by propofol in two studies [11, 25] and sevoflurane with or without N_2_O in the remaining studies [7, 9, 10, 15, 22–24]. Anesthesia was maintained under sevoflurane anesthesia with or without N_2_O in all studies. No study reported total intravenous anesthesia. The surgery types were minor elective surgery [7], elective ambulatory surgery [9, 22], tonsillectomy [15, 25] or adenoidectomy [25], ophthalmic surgery [10], elective infraumbilical surgery [11], and oesophageal dilatation procedures [24]. The diagnosis tools of EA included the Watcha scale [9, 11], pediatric anesthesia emergence delirium scale [10, 15, 25], Aono’s scale [15], Keegan scale [22], five-point scale [23], EA scale [24], and pain/discomfort scale [7]. One study did not provide a cut-off value for the Watcha scale [11]. The corresponding author was consulted by email, and it was determined that the patients were considered to have EA when the Watcha scale score was > 2 in this study.

**Table 1:** Clinical characteristics of the included studies

Study	Sample size	ASA	Age	Sex (M/F)	Type of surgery	Anesthetic agents	Postoperative analgesia	Melatonin dosage	Diagnosis tool for EA	Control	Foundation
Ali [11] 2020	105	1 to 2	3 to 8 years	91/14	Elective infraumbilical surgical procedure	Induce: propofol + fentanyl+atracurium; Maintain: sevoflurane + N_2_O;	Paracetamol	0.2 mg kg^− 1^	Watcha scale	Dexmedetomidine or clonidine	Not mentioned
Jangra [10] 2022	120	1 to 2	3 to 9 years	75/45	Ophthalmic surgery	Induce: sevoflurane+fentanyl; Maintain: sevoflurane + N_2_O;	Fentanyl for postoperative pain rescue	0.5 mg kg^− 1^	PAED scale	Dexmedetomidine	Departmental funds
Kain [22] 2009	148	1 to 2	2 to 8 years	82/66	Outpatient elective surgery	Induce: sevoflurane + N_2_O; Maintain: not mentioned;	Not mentioned	0.05, 0.2, 0.4 mg kg^− 1^	Keegan scale (3-point scale)	Midazolam	Not mentioned
Khalifa [23] 2013	60	not reported	3 to 6 years	31/29	Not reported	Induce: sevoflurane + cisatracurium; Maintain: sevoflurane;	Paracetamol	0.1 mg kg^− 1^	Five-point scale	Placebo (saline)	Not mentioned
Komazaki [15] 2020	48	1 to 2	18 to 119 months	37/11	Tonsillectomy	Induce: sevoflurane + N_2_O +fentanyl+rocuronium; Maintain: sevoflurane;	Paracetamol	0.1 mg kg^− 1^	PAED scale, Aono’s scale	Placebo (syrup)	National Funds
Ozcengiz [24] 2011	100	1 to 2	3 to 9 years	50/50	Oesophageal dilatation procedures	Induce: sevoflurane + N_2_O+vecuronium; Maintain: sevoflurane + N_2_O;	Paracetamol	0.1 mg kg^− 1^	EAS (4-point scale)	Placebo (saline), midazolam, or dexmedetomidine	Not mentioned
Samarkandi [7] 2005	75	1	2 to 5 years	50/25	Minor elective surgery	Induce: sevoflurane + N_2_O; Maintain: sevoflurane + N_2_O;	Paracetamol + caudal block	0.1, 0.25, 0.5 mg kg^− 1^	Pain/discomfort scale (0 to 6 points)	Placebo (acetaminophen)^a^ or midazolam	Departmental funds
Singla [9] 2021	132	1 to 2	3 to 8 years	93/39	Elective ambulatory procedure	Induce: sevoflurane + N_2_O + fentanyl; Maintain: sevoflurane + N_2_O;	Fentanyl or regional block if required	0.3 mg kg^− 1^	Watcha scale	Placebo (honey) or midazolam	Departmental funds
Song [25] 2020	163	1 to 2	2 to 6 years	99/64	Elective adenotonsillectomy or adenoidectomy	Induce: propofol + fentanyl + succinylcholine; Maintain: sevoflurane + N_2_O	Ketorolac	0.5 mg kg^− 1^	PAED scale	Placebo (syrup) or midazolam	Not mentioned

a. In this study, melatonin or midazolam was orally administered mixed with acetaminophen, and the researchers identified oral acetaminophen alone as the placebo comparator

Abbreviations: ASA, American Society of Anesthesiologists; PAED, Pediatric anesthesia emergence delirium; EAS, Emergence Agitation Scale

### Risk of bias in the studies

**Fig. 2:**
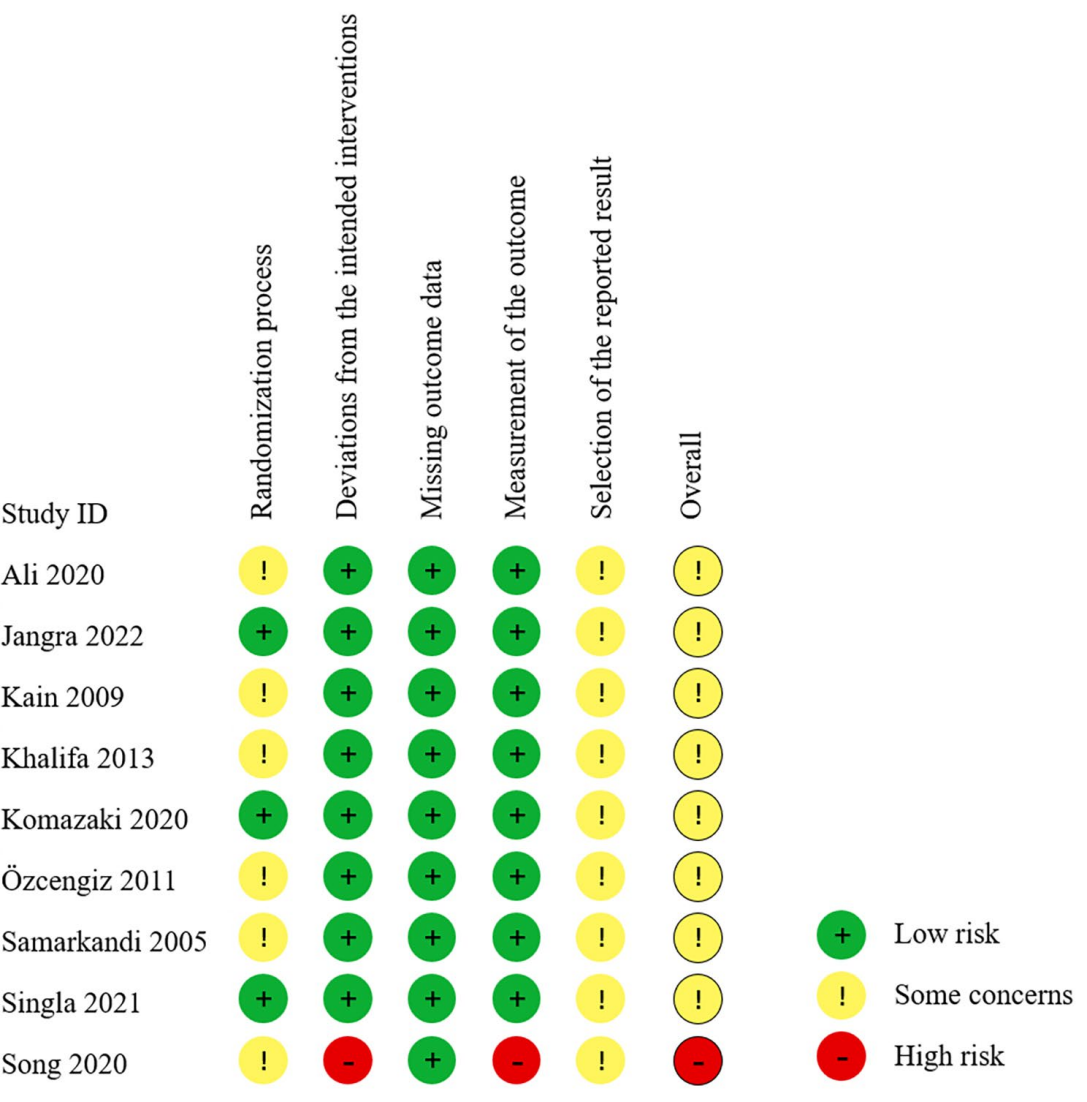
Risk bias of the included studies

The risk of bias is shown in Fig. 2. One study [25] was considered to have a high risk because the researcher reported in the trial registry record that it was an open-label study and the thesis did not provide any blinding information. Six studies [7, 11, 22–25] raised some concerns regarding the randomization process because the allocation concealment was not described. There were some concerns regarding the selection of the reported results in all the studies: prospectively registered protocols were missing in five studies [7, 11, 22–24], and the multiple time points of EA assessment were not mentioned in the registered protocols of the remaining four studies [9, 10, 15, 25].

### Incidence of EA

#### Melatonin or its analogs vs. placebos

**Fig. 3:**
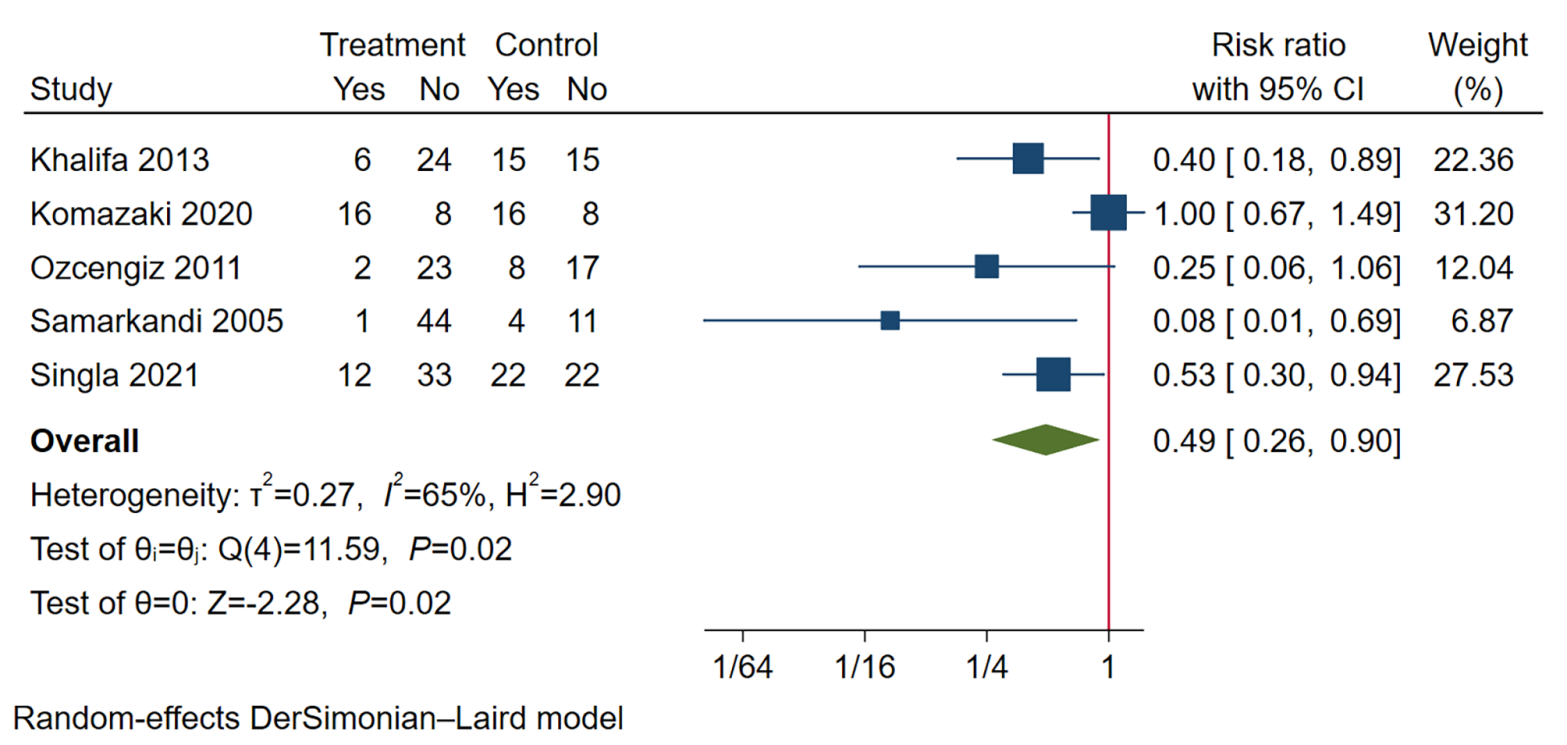
A forest plot comparing the incidence of pediatric emergence agitation between melatonin or its analogs and placebo groups

**Fig. 4:**
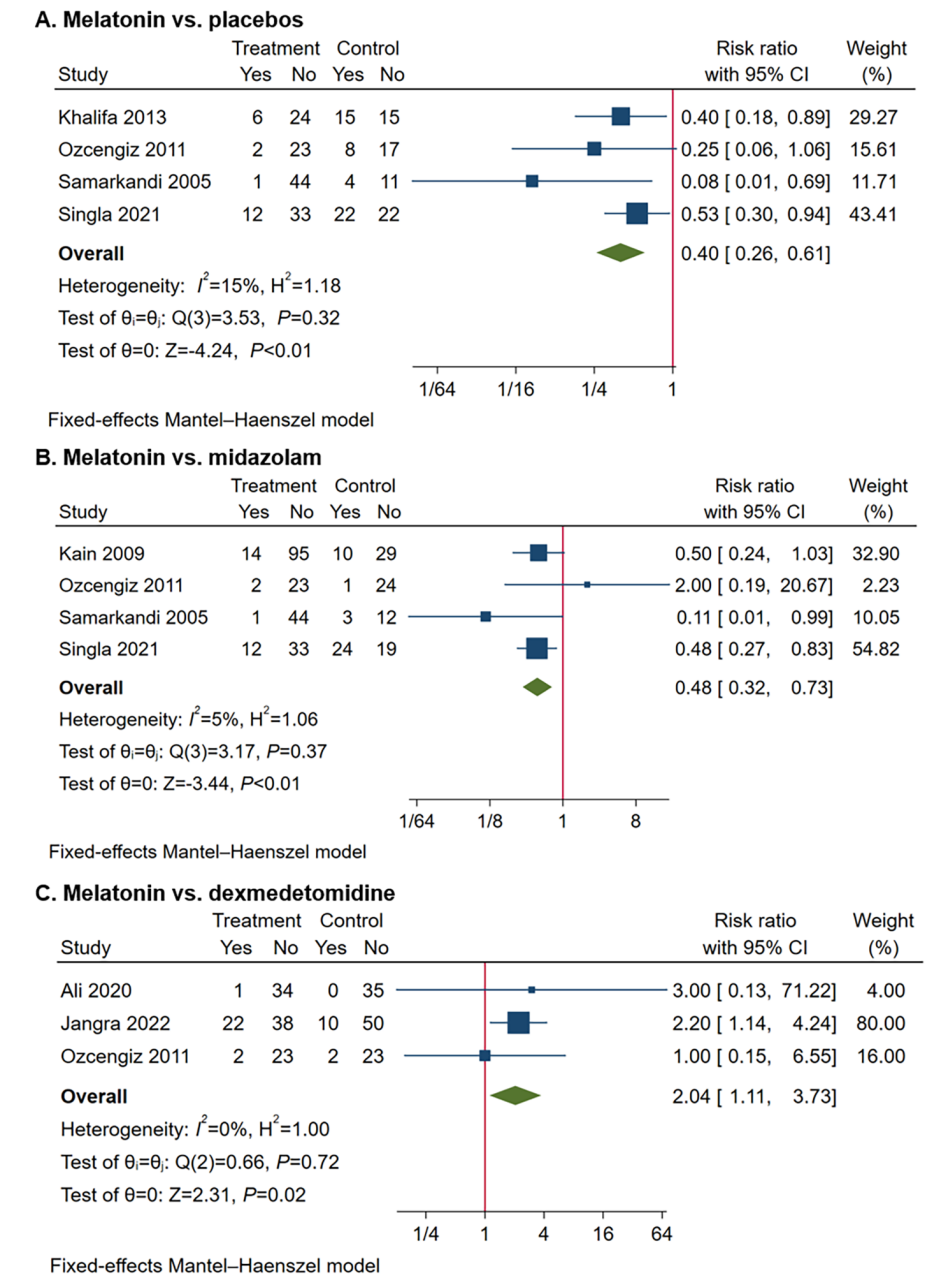
Forest plots comparing the incidence of pediatric emergence agitation between melatonin and control groups. **A**, Melatonin vs. placebos; **B**, Melatonin vs. midazolam; **C**, Melatonin vs. dexmedetomidine

In studies comparing melatonin with placebos, the incidence of EA was 21.9% in the melatonin and its analogs group and 47.1% in the placebos group. Melatonin and its analogs remarkably decreased EA incidence compared with placebos (RR 0.49, 95% CI 0.26 to 0.90, *P* = 0.02; TSA-adjusted CI 0.03 to 7.30; participants *n* = 307; studies *n* = 5) (Fig. 3). In the TSA, the cumulative Z-curve did not pass through the TSA boundary, with 10.7% of RIS cases (*n* = 2873) accrued (eFig. 1 in the Supplement). The statistical heterogeneity was substantial (*I*^*2*^ = 65%, *P =* 0.02). One study [15] using a melatonin analog (ramelteon) instead of melatonin had a major impact on the heterogeneity. Excluding this study obviously reduced the heterogeneity (*I*^*2*^ = 15%, *P* = 0.32) with no change in the meta-analysis results (RR 0.40, 95% CI 0.26 to 0.61, *P* < 0.01; TSA-adjusted CI 0.18 to 0.88; participants *n* = 259; studies *n* = 4) (Fig. 4A). In the TSA of melatonin premedication, the cumulative Z-curve passed through the TSA boundary before reaching the RIS (*n* = 755) (eFig. 2 in the Supplement). In addition, the meta-regression analysis showed no significant effect modification by dose of melatonin compared with placebo (regression coefficient 0.99, 95% CI -3.02 to 5.00, *P* = 0.63).

#### Melatonin vs. midazolam

In studies comparing melatonin with midazolam, the incidence of EA was 12.9% in the melatonin group and 31.1% in the midazolam group. Melatonin significantly decreased EA incidence compared with midazolam (RR 0.48, 95% CI 0.32 to 0.73, *P* < 0.01; TSA-adjusted CI 0.21 to 1.12; participants *n* = 346; studies *n* = 4) (Fig. 4B). In the TSA, the cumulative Z-curve did not pass through the TSA boundary, with 29.4% of RIS cases (*n* = 1175) accrued (eFig. 3 in the Supplement). Heterogeneity was not detected (*I*^*2*^ = 5%, *P* = 0.37). In addition, the meta-regression analysis showed no significant effect modification by dose of melatonin compared with midazolam (regression coefficient − 3.85, 95% CI -7.84 to 0.13, *P* = 0.06).

#### Melatonin vs. dexmedetomidine

In studies comparing melatonin with dexmedetomidine, EA incidence was 20.8% in the melatonin group and 10.0% in the dexmedetomidine group, with a significant difference (RR 2.04, 95% CI 1.11 to 3.73, *P* = 0.02; TSA-adjusted CI 0.17 to 24.04; participants *n* = 240; studies *n* = 3) (Fig. 4C). In the TSA, the cumulative Z-curve did not pass through the TSA boundary, with 6.0% of RIS cases (*n* = 4011) accrued (eFig. 4 in the Supplement). No obvious heterogeneity (*I*^*2*^ = 0%, *P* = 0.72) was found. In addition, the meta-regression analysis showed no significant effect modification by dose of melatonin compared with dexmedetomidine (regression coefficient 1.50, 95% CI -3.13 to 6.12, *P* = 0.53).

#### Melatonin vs. clonidine

Only one study performed this comparison. Hence, no meta-analysis or TSA was performed. Melatonin did not attenuate the incidence of EA compared with clonidine (RR 3.0, 95% CI 0.13 to 71.22, *P* = 0.50). The wide 95% CI reveals the statistical imprecision.

### Adverse effects

One study reported that no melatonin-relevant adverse effects were observed [24]. The corresponding authors of the other studies were contacted by email. Two authors [9, 11] responded that no adverse effects related to melatonin were found. One author [7] responded to a previous meta-analysis [16] stating that no melatonin-related adverse effects were found, but they did not respond to our contact. Two studies [10, 11] focusing on melatonin and dexmedetomidine reported a lower heart rate after dexmedetomidine premedication compared with melatonin. One of these [10] reported that no participants had symptomatic bradycardia requiring pharmacological intervention.

### Reporting biases

The funnel plots of melatonin compared with placebos and midazolam are shown in eFig. 5 in the Supplement. Funnel plots of melatonin compared with dexmedetomidine or clonidine could not be generated because the paucity of studies precluded meaningful analysis. A visual inspection of the melatonin and placebo funnel plots indicated obvious asymmetry, suggesting the existence of publication bias (eFig. 5 A in the Supplement). No publication bias was observed in the melatonin and midazolam funnel plots (eFig. 5B in the Supplement).

### Certainty of evidence

A summary of the findings is presented in Table 2, and the certainty of the evidence was assessed as very low or moderate in all outcomes.

**Table 2:** Summary of the findings

Outcomes	Anticipated absolute effects (95% CI)		Relative effect (95% CI)	№ of participants (studies)	Certainty of the evidence (GRADE)
	Risk with control	Risk with melatonin			
Incidence of EA					
Melatonin and its analogs vs. placebos	471 per 1000	240 fewer per 1000 (from 349 fewer to 47 fewer)	0.49 (0.26 to 0.90)	307 (5)	⨁◯◯◯ Very low ^abc^
Melatonin vs. placebos	430 per 1000	258 fewer per 1000 (from 318 fewer to 168 fewer)	0.40 (0.26 to 0.61)	259 (4)	⨁⨁⨁◯ Moderate ^c^
Melatonin vs. midazolam	311 per 1000	162 fewer per 1000 (from 212 fewer to 84 fewer)	0.48 (0.32 to 0.73)	346 (4)	⨁⨁⨁◯ Moderate ^b^
Melatonin vs. dexmedetomidine	100 per 1000	104 more per 1000 (from 11 more to 273 more)	2.04 (1.11 to 3.73)	240 (3)	⨁⨁⨁◯ Moderate ^b^

a. For high *I*^*2*^ scores, the level of certainty was downgraded to “serious” for “inconsistency.”

b. For the wide range of TSA-adjusted 95% CI, the certainty of evidence was downgraded to “serious” for “imprecision.”

c. Publication bias was strongly suspected

Abbreviations: EA, Emergence agitation; CI, Confidence interval; GRADE, Grading of Recommendations Assessment, Development, and Evaluation

## Discussion

This meta-analysis suggests that the prophylactic use of melatonin significantly decreases EA incidence compared with the use of placebos and midazolam. Dexmedetomidine premedication has a stronger effect than melatonin in preventing EA. The TSA indicates that more RCTs are needed to confirm the findings. No melatonin-related adverse effects were found in this meta-analysis.

A previous meta-analysis reported that melatonin premedication decreased EA incidence in children after general anesthesia [16]. However, that systematic review extended only until April 2014 and considered four RCTs with 358 participants. Nor did it include studies on melatonin analogs, and it was unable to compare melatonin with dexmedetomidine as only one study associated with dexmedetomidine was included. In contrast, our systematic review used a more comprehensive search strategy, including searching for gray literature and undertaking a manual search of reference lists. We updated the search to include papers published until November 2022 and found nine RCTs with 951 participants. The present meta-analysis also used rigorous methodological and quality-of-evidence assessments. Studies with a high risk of bias were excluded from the primary analysis to avoid impairing credibility, reduce the overall bias, and increase the homogeneity of this meta-analysis [26–28].

It is found that melatonin, compared to the use of a placebo, decreases the incidence of pediatric EA after general anesthesia, which is similar to the findings of the previous meta-analysis [16]. Unlike the previous meta-analysis, the TSA in our meta-analysis showed that a significant result has been achieved. However, it is paramount to approach this result with caution. The RIS has yet to be achieved, and thus there is a risk of random errors. Such findings underscore the importance of continuing research comparing melatonin and placebos to ensure the robustness of the observed outcomes.

The present systematic review included one study [15] on a melatonin analog, which reported that 0.1 mg kg^− 1^ ramelteon could not prevent EA in children after general anesthesia. The authors deemed that 0.1 to 0.5 mg kg^− 1^ of melatonin effectively prevented EA in children, and as ramelteon had a higher affinity than melatonin, 0.1 mg kg^− 1^ of ramelteon was chosen. However, higher affinity does not necessarily mean a greater effect [29]. The effects of drugs on the human body are potentially influenced by various factors, including drug efficacy [30] and pharmacokinetic properties [31]. Besides, melatonin exerts its effects via both receptor-dependent and receptor-independent mechanisms [32]. While ramelteon exhibits superior receptor affinity to melatonin, this does not necessarily guarantee enhanced efficacy in preventing EA. Two studies [33, 34] performed on adult participants indicated the preventive effects of ramelteon on delirium. The dose of ramelteon in these two studies was higher than the common dose of melatonin in preventing delirium (8 mg d^− 1^ ramelteon [33, 34] vs. 3 mg d^− 1^ to 5 mg d^− 1^ melatonin [35]). Thus, ramelteon may have the potential to prevent EA in children, but higher doses may be required. Additional studies are evidently needed to confirm the effect of ramelteon and other melatonin analogs in preventing pediatric EA.

To our knowledge, this is the first study to draw tentative conclusions on melatonin premedication compared with midazolam and dexmedetomidine. The previous meta-analysis [16] reached no conclusion on the effects of melatonin compared with dexmedetomidine or midazolam because of the small number of participants included. In our meta-analysis, melatonin showed a greater effect than midazolam, and dexmedetomidine showed a greater effect than melatonin, in preventing EA. However, the results of the comparison between melatonin and midazolam or dexmedetomidine should be interpreted cautiously, as both TSA results suggest that further studies are warranted to confirm the findings.

No adverse effects of melatonin predication were reported in the meta-analysis. According to the previous studies, melatonin appears to be well tolerated and safe at a high dose (10 mg kg^− 1^ [36, 37]) or over a long course [38]. Previous studies reported no addiction or detrimental effect on children’s growth [38]. The possible side effects of melatonin include dizziness, headache, nausea, and sleepiness [39], which were reported in children who made long-term use of melatonin [38]. No studies reported severe adverse effects of exogenous melatonin in children. However, the paucity of complete adverse effects data prevents us from drawing any conclusions regarding the safety of melatonin premedication.

Our review has certain limitations. First, the exclusion of studies with a high risk of bias decreased the sample size, thus reducing the overall precision. Second, although we searched multiple databases and the gray literature without language restriction, the TSA indicates that more RCTs are needed to confirm the findings on melatonin premedication in mitigating EA in pediatric patients. Third, the funnel plots indicated that there was a publication bias in the comparison between melatonin or its analogs and placebos. Finally, our meta-analysis revealed inconsistencies across the included studies, spanning several confounding factors. These inconsistencies encompassed aspects such as placebos, diversity in anesthesia types, surgical procedures, and EA diagnostic tools. Due to the limited number of studies available, we were unable to perform a detailed subgroup analysis to account for these potential confounders. In light of this, the conclusions presented in this paper should be interpreted with caution. We strongly recommend further research on melatonin premedication in pediatric populations with a particular emphasis on an exploration of different anesthesia modalities, surgical interventions, and diagnostic approaches for EA.

## Conclusion

In summary, the prophylactic use of melatonin significantly decreases EA incidence compared with placebos and midazolam. Premedication with dexmedetomidine has a stronger effect than that with melatonin in preventing EA. The results from the TSA suggest that additional research is essential to conclusively determine the efficacy of melatonin premedication in mitigating EA in pediatric patients. More research is necessary to confirm the effect of melatonin analogs in preventing pediatric EA.

### Electronic supplementary material

Below is the link to the electronic supplementary material.

Supplementary Material 1

## Data Availability

All data generated or analyzed during this study are included in this published study [and its supplementary information files].

## Abbreviations

EA: Emergence agitation
EMBASE: Excerpta Medica Database
CNKI: China National Knowledge Infrastructure
ASA: American Society of Anesthesiologists
RR: risk ratio
CI: confidence interval
TSA: Trial sequential analysis
RIS: required information size
GRADE: Grading of Recommendations Assessment, Development and Evaluation
