# Supplementary material for: Melatonin or its analogs as premedication to prevent emergence agitation in children: a systematic review and meta-analysis
Source: BMC Anesthesiol. 2023 Nov 30;23:392. doi: 10.1186/s12871-023-02356-x (PMC10687973; doi:10.1186/s12871-023-02356-x)
Supplement: Supplementary file 1 — Supplementary Material 1 [file 12871_2023_2356_MOESM1_ESM.docx]

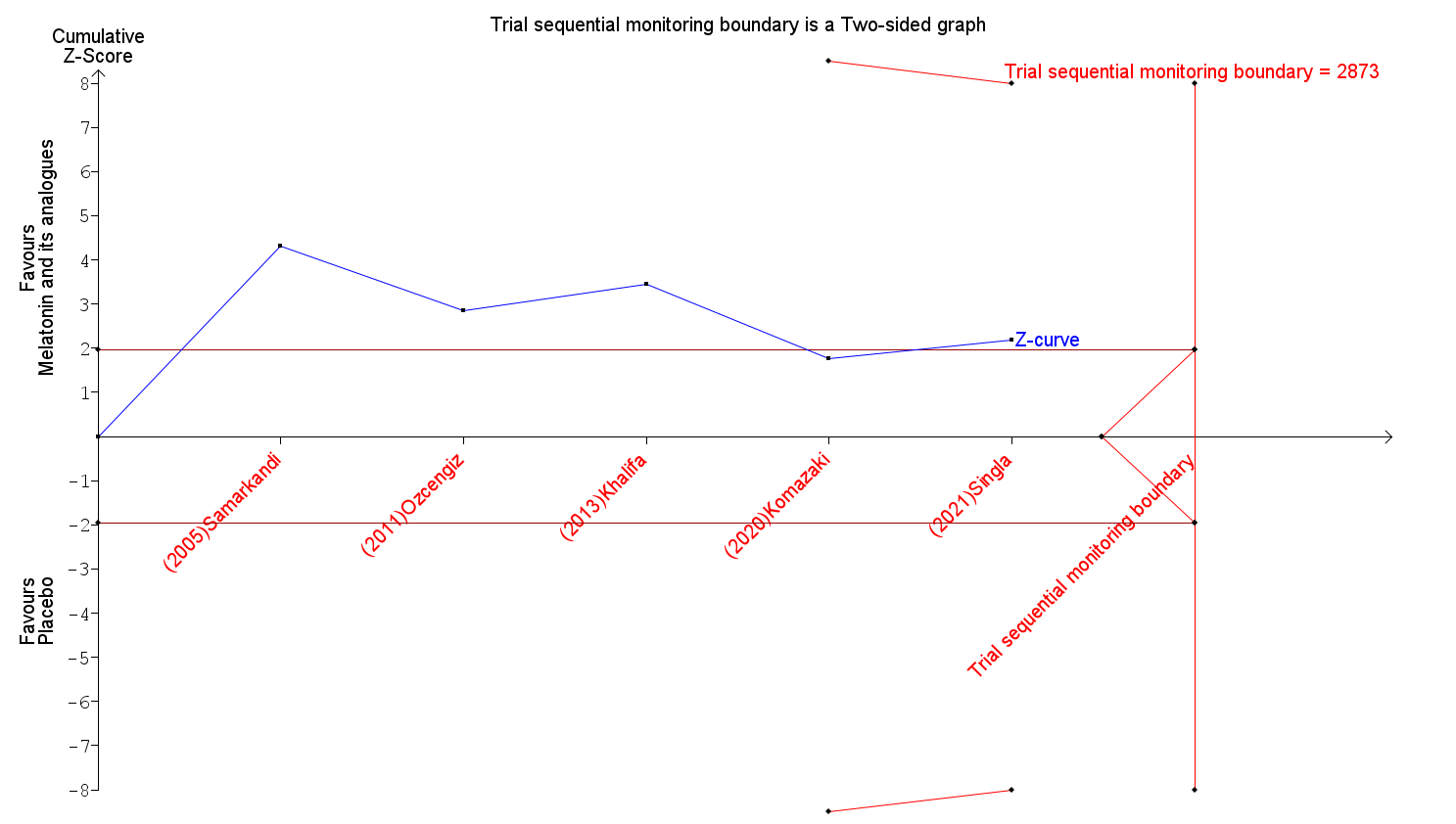


eFig. 1. Trial sequential analysis of comparing the incidence of pediatric emergence agitation between melatonin or its analogs and placebo groups.


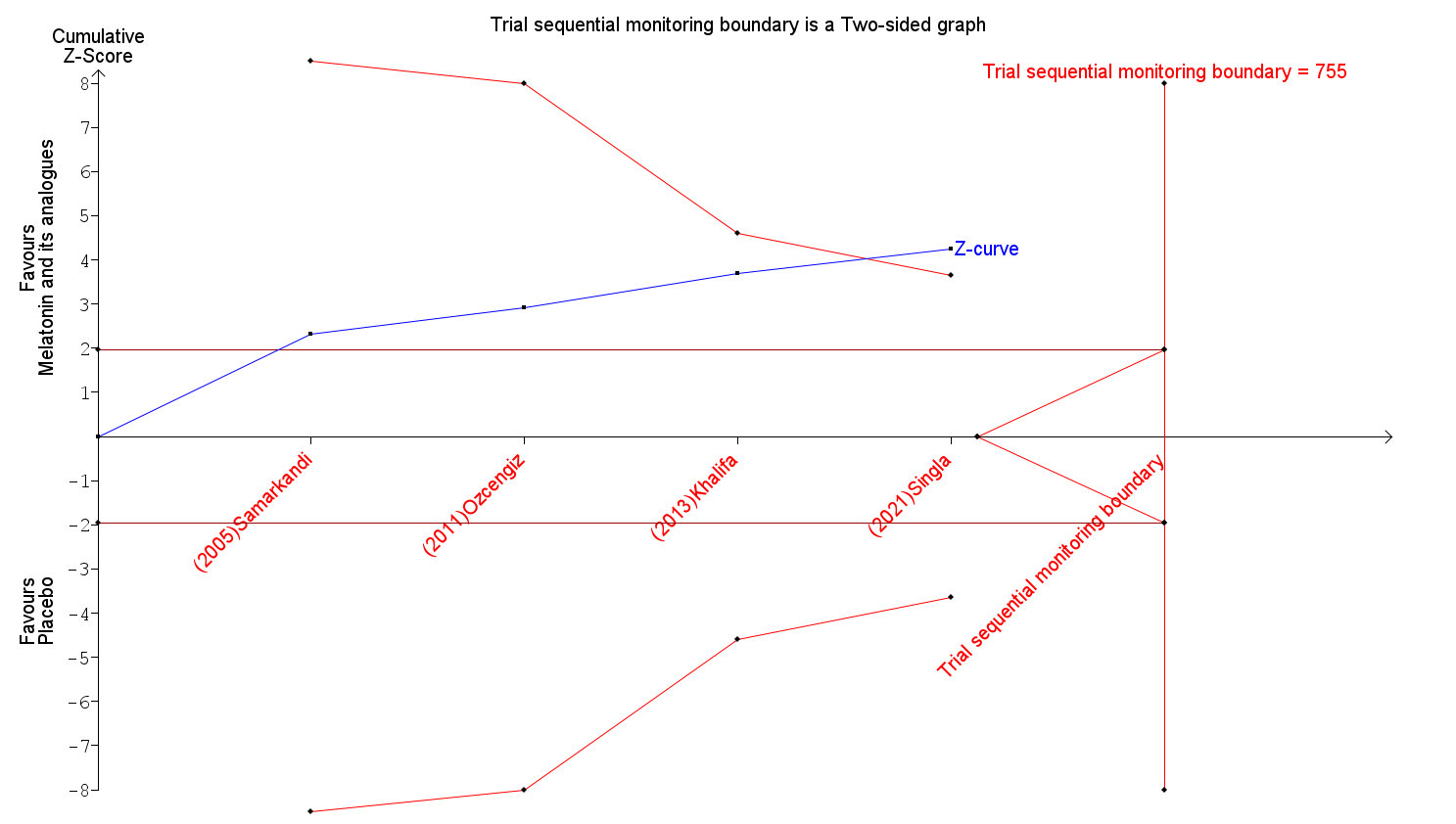
 eFig. 2. Trial sequential analysis of comparing the incidence of pediatric emergence agitation between melatonin and placebo groups.


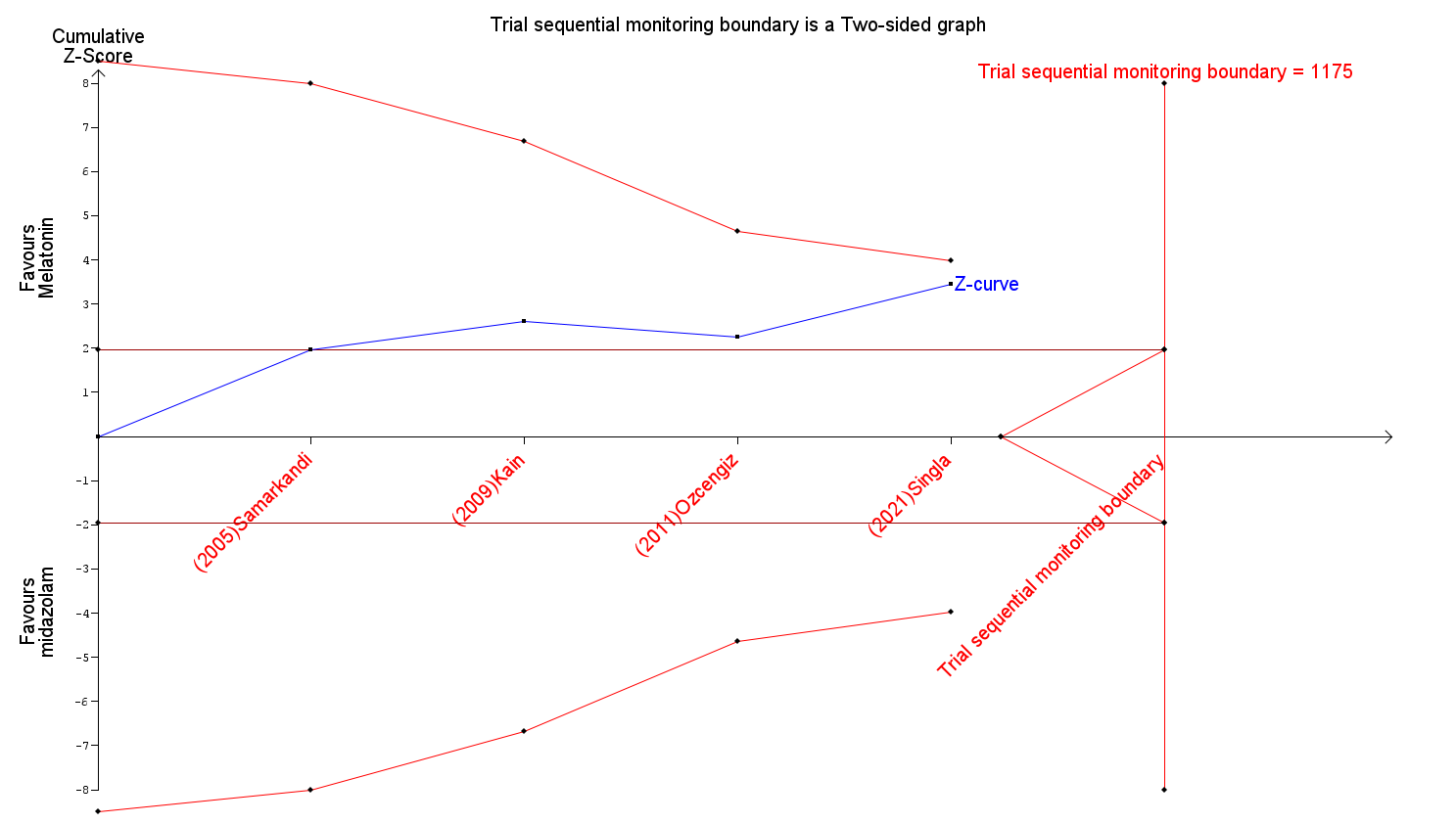


eFig. 3. Trial sequential analysis of comparing the incidence of pediatric emergence agitation between melatonin and midazolam groups.


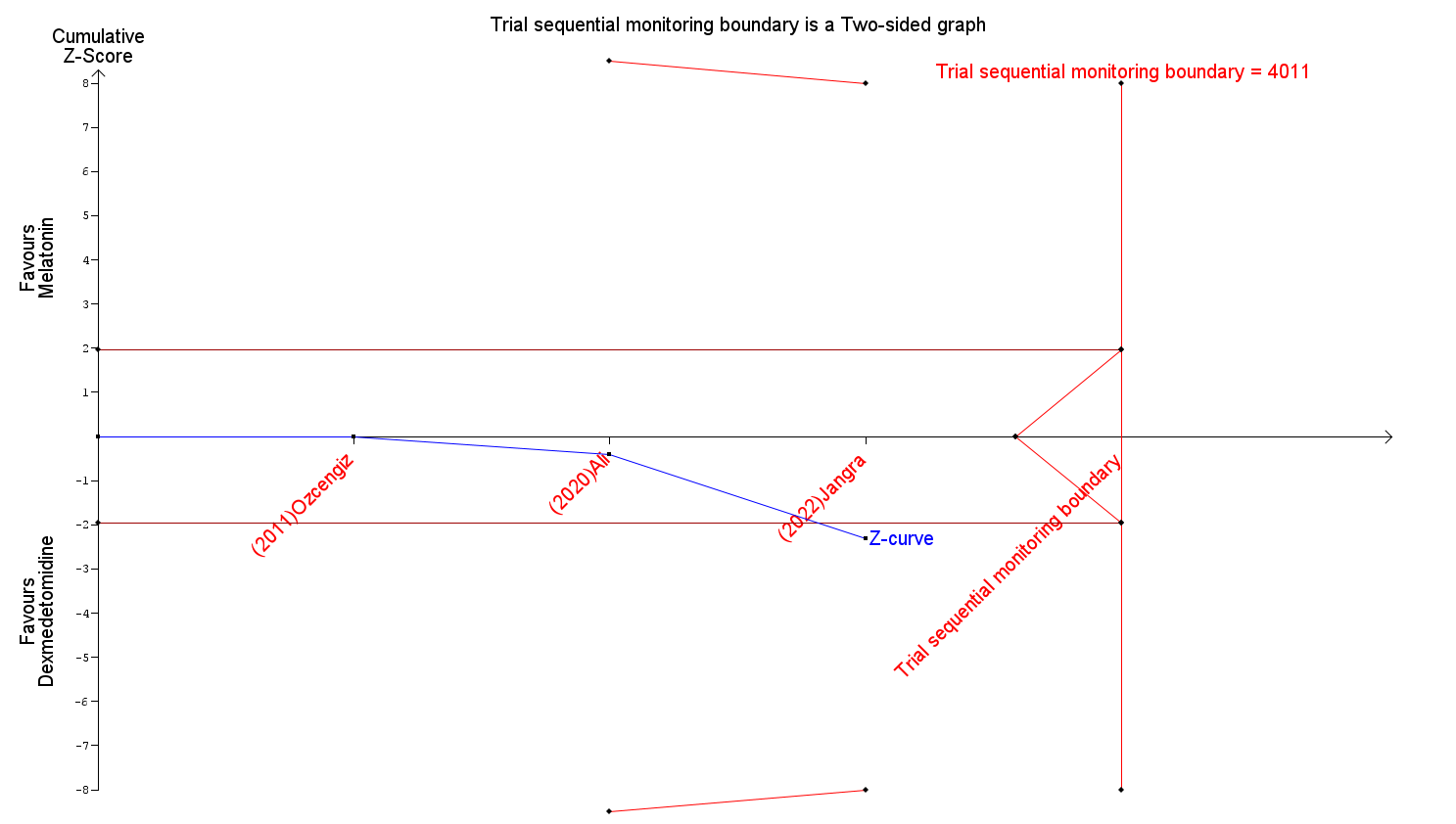


eFig. 4. Trial sequential analysis of comparing the incidence of pediatric emergence agitation between melatonin and dexmedetomidine groups.


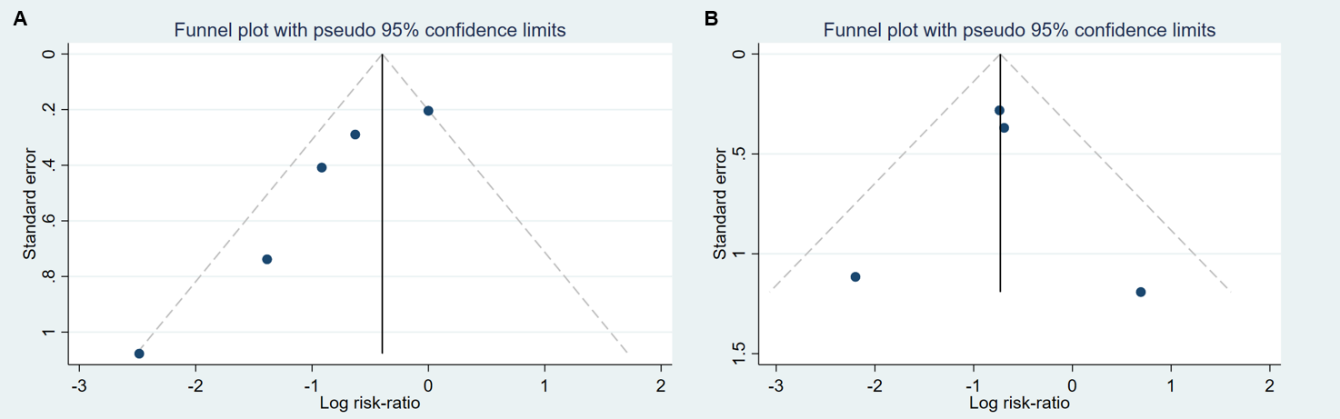


eFig. 5. Funnel plots of emergence agitation incidence. A, Melatonin or its analogs vs. placebo; B, Melatonin vs. midazolam.

eTable 1. Search strategy for each database.

| Database | Order/items | Keywords | Results |
| --- | --- | --- | --- |
| PubMed | #1 | "melaton*"[All Fields] OR "ramelteon"[Supplementary Concept] OR "ramelteon"[All Fields] OR "rozerem"[All Fields] OR "tak 375"[All Fields] OR "tasimelteon"[Supplementary Concept] OR "tasimelteon"[All Fields] OR "agomelatin*"[All Fields] OR "valdoxan"[All Fields] OR "s 20098"[All Fields] OR "melaxen"[All Fields] | 31379 |
|  | #2 | "delirium*"[All Fields] OR "agitat*"[All Fields] | 46,268 |
|  | #3 | #1 AND #2 | 324 |
| EMBASE | #1 | melaton* OR 'ramelteon'/exp OR 'ramelteon' OR 'rozerem'/exp OR 'rozerem' OR 'tak 375'/exp OR 'tak 375' OR 'tasimelteon'/exp OR 'tasimelteon' OR agomelatin* OR 'valdoxan'/exp OR 'valdoxan' OR 's 20098'/exp OR 's 20098' OR 'melaxen'/exp OR 'melaxen' | 48740 |
|  | #2 | delirium* OR agitat* | 91830 |
|  | #3 | #1 AND #2 | 1065 |
| Web of science | #1 | ((((((((ALL=(Melaton*)) OR ALL=(ramelteon)) OR ALL=(rodegem)) OR ALL=(tasimelteon)) OR ALL=(agomelatin*)) OR ALL=(valdoxane)) OR ALL=(melafen)) OR ALL=(tak 375)) OR ALL=(s 20058) | 33,941 |
|  | #2 | (ALL=(delirium*)) OR ALL=(agitat*) | 52,757 |
|  | #3 | #1 AND #2 | 466 |
| Cochrane | #1 | (Melaton*) OR (ramelteon) OR (rozerem) OR (tak 375) OR (tasimelteon) OR (agomelatin*) OR (valdoxan) OR (s 20098) OR (melaxen) | 4273 |
|  | #2 | (delirium*) OR (agitat*) | 10481 |
|  | #3 | #1 AND #2 | 342 |
| Wanfang | #1 | 全部:(褪黑素) OR 全部:(美拉通宁) OR 全部:(雷美替胺) OR 全部:(瑞美替胺) OR 全部:(雷美尔通) OR 全部:(拉米替隆) OR 全部:(他司美琼) OR 全部:(阿戈美拉汀) OR 全部:(维度新) OR 全部:(烦多闪) | 232204 |
|  | #2 | 全部:(躁动) OR 全部:(谵妄) | 35041 |
|  | #3 | #1 AND #2 | 96 |
| CNKI | #1 | TKA='褪黑素' OR TKA='美拉通宁' OR TKA='雷美替胺' OR TKA='瑞美替胺' OR TKA='雷美尔通' OR TKA='拉米替隆' OR TKA='他司美琼' OR TKA='阿戈美拉汀' OR TKA='维度新' OR TKA='烦多闪' | 6491 |
|  | #2 | TKA='躁动' OR TKA='谵妄' | 28409 |
|  | #3 | #1 AND #2 | 48 |
| ProQuest Dissertations & Theses Global | #1 | noft(Melaton* OR ramelteon OR rozerem OR (tak 375) OR tasimelteon OR agomelatin* OR valdoxan OR (s 20098) OR melaxen) | 1,269 |
|  | #2 | noft(delirium OR agitat*) | 5,547 |
|  | #3 | #1 AND #2 | 5 |
| Clinicaltrials | Condition or disease | delirium OR deliriums OR agitate OR agitated OR agitates OR agitating OR agitation OR agitations OR agitator OR agitators | 52 |
|  | Other terms | melatonin OR melatonine OR melatonins OR ramelteon OR rozerem OR (tak 375) OR tasimelteon OR agomelatine OR valdoxan OR (s 20098) OR agomelatin OR melaxen OR melatonergics |  |
|  | Study type | All studies |  |
|  | Study Results | All studies |  |
| WHO ICTRP | in the title |  | 62 |
|  | in the condition | (delirium*) OR (agitat*) |  |
|  | in the intervention | (Melaton*) OR (ramelteon) OR (rozerem) OR (tak 375) OR (tasimelteon) OR (agomelatin*) OR (valdoxan) OR (s 20098) OR (melaxen) |  |
| Google |  | (melatonin OR melatonine OR ramelteon OR rozerem OR tasimelteon OR agomelatine OR valdoxan OR melaxen OR melatonergics) AND (delirium OR agitate OR agitating OR agitation) | 22100  (first 300 were screened) |
